# Determinants of awareness, value perception, and societal support for sexual and reproductive health services among in-school adolescents in South-eastern Nigeria

**DOI:** 10.1186/s12913-023-09470-z

**Published:** 2023-05-17

**Authors:** Irene Ifeyinwa Eze, Chinyere Ojiugo Mbachu, Ifunanya Clara Agu, Ifeyinwa Chizoba Akamike, Godstime Eigbiremolen, Obinna Onwujekwe

**Affiliations:** 1 Department of Community Medicine, College of Health Sciences, Alex Ekwueme Federal University Teaching Hospital, Abakaliki, Nigeria; 2 Health Policy Research Group, University of Nigeria, Enugu Campus, Enugu, Nigeria; 3 University of Nigeria, Enugu Campus, Enugu, Nigeria

**Keywords:** Adolescent, Sexual and reproductive health, Service utilisation, Student, Awareness, Perception, Community support, Nigeria

## Abstract

**Background:**

Adolescents are vulnerable to sexual and reproductive health (SRH) risks yet, have poor utilisation of SRH services due to personal, social, and demographic influences. This study aimed to compare the experiences of adolescents that had received targeted adolescent SRH interventions and those that did not and evaluated the determinants of awareness, value perception, and societal support for SRH service utilisation among secondary school adolescents in eastern Nigeria.

**Methods:**

We undertook a cross-sectional study of 515 adolescents in twelve randomly selected public secondary schools, grouped into schools that had received targeted adolescent SRH interventions and those that did not, across six local government areas in Ebonyi State, Nigeria. The intervention comprised training of schools’ teachers/counsellors and peer educators and community sensitisation and engagement of community gatekeepers for demand generation. A pre-tested structured questionnaire was administered to the students to assess their experiences with SRH services. Categorical variables were compared using the Chi-square test, and predictors were determined through multivariate logistic regression. The level of statistical significance was determined at p < 0.05 and a 95% confidence limit.

**Results:**

A higher proportion of adolescents in the intervention group, 126(48%), than in the non-intervention group, 35(16.1%), were aware of SRH services available at the health facility (p-value < 0.001). More adolescents in the intervention than the non-intervention group perceived SRH services as valuable– 257(94.7%) Vs 217(87.5%), p-value = 0.004. Parental/community support for SRH service utilisation was reported by more adolescents in the intervention group than in the non-intervention group- 212 (79.7%) Vs 173 (69.7%), p-value = 0.009. The predictors are (i) awareness-intervention group (β = 0.384, CI = 0.290–0.478), urban residence (β=-0.141, CI=-0.240-0.041), older age (β-0.040, CI = 0.003–0.077) (ii) value perception - intervention group (β = 0.197, 0.141–0.253), senior educational class (β = 0.089, CI = 0.019–0.160), work-for-pay (β=-0.079, CI=-0.156–0.002), awareness (β = 0.192, CI = 0.425–0.721) (iii) parental/community support - work-for-pay (β = 0.095, CI = 0.003–0.185).

**Conclusions:**

Adolescents’ awareness, value perception, and societal support for sexual and reproductive health services were influenced by the availability of SRH interventions and socio-economic factors. Relevant authorities should ensure the institutionalisation of sex education in schools and communities, targeting various categories of adolescents, to reduce disparity in the utilisation of sexual and reproductive health services and promote adolescents’ health.

## Introduction

Adolescents have unique and special needs, including sexual and reproductive health, which are often underserved in many societies. [1] These needs result from physical, psychological, emotional, and social maturation and change from childhood to adulthood. [2] The changes are often accompanied by social and behavioural dilemmas, resulting in poor health risks and outcomes if not well attended to. [3] Thus, adolescents remain vulnerable to sexual and reproductive health (SRH) risks, including early and unwanted pregnancy, unsafe abortions, and sexually transmitted infections. [3]

Most adolescent pregnancies result from the early sexual debut, early marriage, and an unmet need for contraception, which is of great concern, especially in sub-Saharan Africa. [4, 5] In Nigeria, sexually active unmarried women aged 15–19 years have a contraceptive prevalence rate for any method of 28.3%, unmet need of 5.7% and demand for contraception of 8.6% despite contraceptive knowledge being virtually universal (98%).^6^ These factors contribute to teenage pregnancies and childbirth with consequent pregnancy-related complications, which are, in turn leading causes of death among adolescent females aged 15–19 years. [4] Furthermore, childbearing during adolescence is known to cause adverse social consequences, particularly regarding educational attainment, as women who become mothers in their teens are more likely to drop out of school. [6] These highlight the importance of SRH services to protect adolescents’ health.

The World Health Organization (WHO) stated that the key to improving access to SRH services for young people is making SRH services “adolescent-friendly”. [7] Adolescent-friendly health services (AFHS) are defined as services that are accessible, acceptable, equitable, appropriate, and effective for adolescents. [7] In other words, these health services should not restrict adolescents but guarantee confidentiality, respect, and non-judgmental, and are within easy reach and affordable for adolescents. [7] AFHS are meant to attract young people and increase access and uptake. To ensure the delivery of AFHS, countries, including Nigeria, have incorporated these features into their health and education systems to improve the arrangement, provision, and quality of adolescent sexual and reproductive health (ASRH) services. [8–10]

Evidence supports the potential effectiveness of multicomponent interventions incorporating demand creation for adolescent SRH (ASRH) services. [11] However, adolescents still face obstacles in using SRH services, including lack of awareness, poor quality of SRH services, lack of privacy and confidentiality, judgmental attitude of health providers, and poor infrastructures. [12–17] In Nigeria, complex social, interpersonal, and cultural factors contribute to these challenges. [18–19] For instance, healthcare providers determine clients’ suitability for SRH services in health facilities based on their moral values. [18] Furthermore, cultural norms and beliefs about sexuality impact adolescents’ support in accessing and utilising SRH services. [19] Although some studies reported that factors like educational level affects awareness and access to SRH services, there is limited evidence on the social and demographic factors influencing adolescents’ utilisation of SRH services. [12, 20]

Evaluating adolescents’ awareness and perceived value for SRH and societal support for their SRH service utilisation will enable more vigorous enforcement of the child rights act regarding the right to health and well-being. [21, 22] Generating evidence on the social and demographic factors influencing adolescents’ utilisation of SRH services will enable appropriate and targeted options to be advocated to improve access and address disparities. This paper aimed to compare the experiences of adolescents that had received targeted adolescent SRH interventions and those that did not and evaluated the determinants of awareness, perceived value, and parental/community support for SRH service utilisation among secondary school adolescents in Ebonyi state, Nigeria.

## Methods

### Study setting

The study was conducted in secondary schools in Ebonyi state, southeast Nigeria. The state is divided into three zones and has thirteen local government areas (LGAs). Most of the populace (over 75%) reside in rural areas. The state has a total of 415 secondary schools (made up of junior secondary schools (JSS) and senior secondary schools (SSS) and comprising 233 public schools and 182 private schools. [23] Like other states in southeast Nigeria, Ebonyi has poor adolescent sexual and reproductive health indices- contraceptive prevalence rate for any method of 8.2% and total demand for family planning of 31.2% (the least among the south-east states) and an unmet need for family planning of 23.0%.^6^

### Study design

A comparative cross-sectional study was conducted among in-school adolescents from September to December 2021.

### Study participants

The study populations were adolescent males and females aged 13–19 years who were attending secondary schools (junior secondary and senior secondary levels) located in selected communities/LGAs in the state. Participants who declined consent or were unfit to participate due to medical conditions like neurocognitive impairments and disabilities (e.g., deafness) that hinder communication were excluded from the study.

### Sample size and sampling methods

The sample size was calculated using the formula for cross-sectional studies. [24] Assuming a confidence interval of 95%, power of 80%, a prevalence of 55% (awareness)^25^ and non-response of 10%, a minimum sample size of 458 was estimated. However, data were collected from 514 adolescents to account for any incomplete responses or incorrectly filled questionnaires.

A multi-stage (three-stage) sampling method was used to recruit participants for the study. In the first stage, the state was stratified into three senatorial zones. From each zone, two LGAs were purposively selected based on geographic location, one urban and one rural, and teenage pregnancy rates. In the second stage, two public secondary schools were selected from each LGA; random selection by balloting schools that had not received targeted SRH intervention and purposive selection of those that had received the intervention, totalling 12 schools. The multi-faceted intervention comprised training of schools’ teachers/counsellors and peer educators and community sensitisation and engagement of community gatekeepers for demand generation and buy-in of the programme. In the final stage, 42 to 43 students were consecutively recruited from each school. Proportionate numbers of students were recruited from each level of study.

### The SRH intervention

The intervention was designed to address the SRH needs of adolescents and improve their access to SRH information and services. The intervention was multi-faceted, comprising (i) a three-day residential training workshop of 29 state trainers to raise a critical mass of competent and skilled trainers to train other providers, (ii) three-day training of 22 school teachers (including principals and guidance counsellors)), (iii) two-day training of 22 peer mentors (iv) establishment and inauguration of school-based youth health clubs, (v) distribution of SRH customized educational items- notepads, fliers shirts, caps, wrist bands, pens), vi). Supportive supervision, and vii) community sensitisation and engagement of 20 community gatekeepers from each selected community.

The participants in the training included the school principals, biology teachers, health education teachers, guidance counsellors (G&C) and senior secondary students purposively selected from six government schools located in the participating communities. The training was delivered using a manual adapted from the national guideline and modified to suit the context. The manual consists of eight modules: (i) Introduction to adolescence and adolescent health; (ii) sexuality and sexual behaviours; (iii) sexually transmitted infections; (iv) principles and practice of counselling; (v) Pregnancy and prevention of pregnancy; (vi) counselling practices on selected health issues of adolescents; (vii) optimal adolescent and youth-friendly services; and (viii) record-keeping and health information systems and (ix) principles and practice of counselling. The training was facilitated by five researchers and two boundary partners and delivered using multiple formats - lecturers, PowerPoint presentations, flip charts, demonstration, roleplay, and discussion, Further details of the intervention can be found in an earlier published manuscript. [25]

**Data Collection**: The data collection instrument was a structured questionnaire adapted from the World Health Organization’s (WHO) illustrative questionnaire for surveys with young people. [26] Forty-two research assistants, both males and females, were trained for four days on the study’s objectives, data collection techniques, and ethics in research. The questions focused on adolescent experiences with SRH services and had four sections: -i) socio-demographic characteristics, ii) awareness of SRH services, iii) perceived value for SRH service, and iv) parental and community support for using SRH services. The questionnaire was pretested on fifty students in another school not selected for the study to ensure validity.

Trained research assistants assisted in the collection of data using the pre-tested questionnaire. The respondents were administered the questionnaires in their schools by the research assistants after getting informed written consent/assent from the school authorities and individual respondents. Paper and electronic (Kobo collect) copies of the questionnaires were used to collect data over two weeks. Electronic copies of the questionnaires were uploaded to android tablets from kobo collect. Individual matching of information on the completed paper questionnaire with corresponding electronic questionnaires was carried out before and after uploading the data to the server.

### Ethical considerations

Ethical approval was obtained from the Research and Ethics Committees of the University of Nigeria Teaching Hospital Enugu (Ref: UNTH/CSA/329/OL.5) and Ebonyi State Ministry of Health (Ref: ERC/SHOH/AI/050/18). Approvals/permission were obtained from the school principals. Informed written consent was obtained from parents/guardians of adolescents aged 13 to 17 and older adolescents aged 18 years and above. Furthermore, a written accent was obtained from adolescents aged 13 to 17. Consent was obtained by signing the consent form after a thorough explanation, including the benefits and risks of participation, was given, and understanding was established. Participation was voluntary, and respondents were informed that they were at liberty to decline to participate or withdraw from the study with no consequences to them at any time. Confidentiality was assured to participants, and personally identifiable information was not captured. The interviews were held in private and convenient locations.

### Data management

#### Description of variable

The variables comprised the independent and dependent/outcome variables.

The independent variables comprised the study groups (intervention or non-intervention group), gender, level of education, area of residence (whether urban or rural), age, whether an individual works for pay, and whether an individual lives with the parent. The outcome variables comprised awareness, perceived value, and societal support for SRH service. The outcome variables were computed as a composite score derived by summing up the scores of the responses from the questions assessing each of the outcome variables- awareness, perceived value, and societal support.

Awareness was determined as a composite score of four variables: (i) awareness of what days/times the health facility is open, (ii) awareness of what SRH services are, (iii) awareness of reproductive health services that are available to adolescents in the health facility and (iv) awareness of SRH services provided in the community.

Perceived value was determined as a composite score of two variables (i) perception that adolescents need SRH services and (ii) perception that adolescents should be provided with reproductive health services.

Societal support was determined as a composite score of three variables (i) parents/guardians support adolescents coming to a health facility for reproductive health services, (ii) there are health services that parents/guardians may not support to be provided to adolescents, and (iii) community members (adult in the community) are supportive of adolescents coming to health facilities for SRH services.

#### Data Analysis

Data was analysed using the STATA standard edition 17 software, Texas, USA. We employed both descriptive and multivariate linear regression models in the study. The descriptive statistics utilised mean, frequency, and percentage. The bivariate analysis utilised the Chi-square test and t-test to determine the differences in the sociodemographic characteristics across the two groups and the differences in the outcome variables -awareness, perceived value, and societal support for SRH service across the two groups. All variables were imputed into a linear regression model- univariable and multivariable regression analysis. Our linear regression model allowed us to take our analysis further by isolating specific predictors of experiences with SRH service (awareness, perceived value, and societal support) while considering variations in individual socio-demographic characteristics under a regression framework. More formally, our multivariate regression model can be specified parsimoniously as.

**γ**_**i**_**= β**_**0**_**+ β**_**1**_**χ**_**i**_**+ µ**_**i**_.

Where **γ**_**i**_ represents the outcome variable for individual *i*. The outcomes of interest include adolescent’s awareness (measured as a composite score of awareness using available SRH awareness variable), adolescent’s perceived value (measured as a composite score of adolescent value of the need and provision of SRH service using available SRH value variables), and societal support for adolescents’ utilisation of SRH services (measured as a composite score of the support adolescent receives from parents and adults in the community for SRH service utilisation using available SRH support variables). **χ**_**i**_ is a vector of control variables for individual i, which includes gender, level of education, area of residence (whether urban or rural), age, whether an individual works for pay or not, whether an individual lives with the parent, and whether an individual belongs to the intervention group or not. The error term, **µ**_**i**_ is taken to be normally distributed. The level of statistical significance was determined by a p-value < 0.05 and a confidence interval of 95%.

## Results

Table 1 shows the socio-demographic characteristics of the adolescents. A total of 514 study participants (266 in the intervention group and 248 in the non-intervention group) took part in the study. The mean age of respondents was 15.6 ± 1.5 years in the intervention group and 15.3 ± 1.5 years in the non-intervention group. Most students were females, the intervention group 72.2%, and the non-intervention group 64.1%. About 65.1% of respondents in the intervention group and 60.0% in the non-intervention group were in senior secondary education. Most students live with their parents/guidance, 93.9% and 94.8% in the intervention and non-intervention groups, respectively. There was no statistically significant difference between the two groups in all the socio-demographic variables.

**Table 1:** Comparison of Socio-demographic characteristics of the adolescents in the intervention and non-intervention group

Variables	Intervention group	Non-Intervention group	*x*2 (*P)*
	Frequency (%) n = 266	Frequency (%) n = 248	
**Location**			
Rural	127 (49.42)	125 (50.81)	0.098 (0.754)
Urban	130 (50.58)	121 (49.19)	
**Gender**			
Female	192 (72.18)	159 (64.11)	3.857 (0.051)
Male	74 (27.82)	89 (35.89)	
**Age group**			
Early adolescent	32 (12.03)	39 (15.73)	3.401 (0.183)
Middle adolescent	199 (74.81)	187 (75.40)	
Late adolescent	35 (13.16)	22 (8.87)	
**Level of schooling**			
Junior Secondary	93 (34.96)	99 (39.92)	1.347 (0.246)
Senior Secondary	173 (65.04)	149 (60.08)	
**Live with parent/guardian**			
No	16 (6.06)	13 (5.24)	0.160 (0.689)
Yes	248 (93.94)	235 (94.76)	
**Work for pay**			
No	214 (81.06)	206 (83.06)	0.348 (0.555)
Yes	50 (18.94)	42 (16.94)	
**Religious affiliation**			
Christian Roman Catholic	106 (39.85)	91 (36.99)	1.66 (0.436)
Christian protestants	160 (60.15)	155 (63.01)	
**Age (mean**±**SD) years**	15.60 (1.55)	15.29 (1.52)	2.3145 (0.9724) ^

^Student’s t-test; SD standard deviation; Early adolescent ≤13years, Middle adolescent >13years<18years, Late adolescent ≥18years.

Table 2 summarises the findings of adolescents’ awareness, value perception, and societal support for the utilisation of SRH services. A significantly higher proportion of the respondent in the intervention group (72.9%) compared to the non-intervention group (50.8%) were aware of SRH services (p < 0.0001). A higher proportion of the respondent in the intervention group was aware of the SRH services available at the facility and in the community than in the non-intervention group. The difference was statistically significant (p < 0.001).

**Table 2:** Association between Adolescents’ awareness, value perception, and societal support with sexual and reproductive health services

Variables	Intervention group (n = 266)	Non-Intervention group (n = 248)	*x*2 (*P)*
	Frequency(%)	Frequency(%)	
**Awareness of available SRH services**			
Aware of what days/times the health facility is open (Yes)	142 (53.58)	106 (42.91)	5.827 (0.016) *
No	124 (46.42)	142 (57.09)	
Aware of what sexual and reproductive health services are (Yes)	194 (72.93)	126 (50.81)	26.739(< 0.0001)*
No	72 (27.07)	122 (49.19)	
Aware of reproductive health services that are available to adolescents in the health facility (Yes)	121 (45.49)	40 (16.13)	51.427(< 0.0001)*
No	145 (54.51)	208 (83.87)	
Aware of SRH services provided in the community (Yes)	126 (47.55)	35 (14.17)	66.066(< 0.0001)*
No	140 (52.45)	213 (85.83)	
**Perceived value for SRH**			
Adolescents need sexual and reproductive health services (Yes)	252 (94.74)	217 (87.50)	8.414 (0.004) *
No	14 (5.26)	31 (12.50)	
Adolescents should be provided with reproductive health services (Yes)	257 (96.62)	222 (89.52)	10.196 (0.001) *
No	9 (3.38)	26 (10.48)	
**Parental/community support**			
Parents support your coming to the health facility for reproductive health services (Yes)	212 (79.70)	173 (69.76)	6.747 (0.009) *
No	54 (20,30)	75 (30.24)	
There are health services that your parents might not want to be provided to you (Yes)	164(62.10)	155(62.50)	0.007 (0.930)
No	102 (37.90)	93 (37.50)	
Adults in the community support adolescent coming to health facility for SRH services (Yes)	210 (79.25)	181 (72.98)	2.770 (0.096)
No	56 (20.75)	67 (27.02)	

* Significant (p < 0.05), SRH sexual and reproductive health,

A significantly higher proportion of the respondent in the intervention group (> 90%) compared to the non-intervention group (> 80%) perceived that adolescents need SRH services (p = 0.004) and that adolescents should be provided with SRH services (p = 0.001). Parent/guardian support to access SRH services at health facilities was significantly higher among the intervention group (79.9%) compared to the non-intervention group (69.7%) with p = 0.009.

Table 3 shows that study group, age and location are predictors of awareness of available SRH services. Being in the intervention group increases adolescents’ awareness of available SRH services by 38% (β = 0.38, CI: 0.290–0.478) as compared to the non-intervention group. For every year increase in age among the participants, there was a 4% increase in awareness of available SRH services (β = 0.04, 0.003–0.077). Living in an urban area decreases awareness of available SRH services by 14.1% (β=-0.141, CI: -0.240 - -0.041) as compared to living in rural areas.

**Table 3:** Linear regression analysis of socio-demographic correlates of awareness of sexual and reproductive health services

Variable	Univariable regression			Multivariable regression		
	Crude Coefficient (β)	P-value	Confidence interval	Adjusted Coefficient (Adjusted β)	P-value	Confidence interval
**Study group**						
Non-intervention group	0.000	Reference	Reference	0.000	Reference	Reference
Intervention group	0.394	< 0.0001	0.299–0.489	0.384	< 0.0001	0.290–0.478
**Gender**						
Male	0.000	Reference	Reference	0.000	Reference	Reference
Female	-0.035	0.524	-0.144-0.074	-0.024	0.638	-0.128- 0.078
**Age**	0.064	< 0.0001	0.032–0.097	0.040	0.033	0.003–0.077
**Educational Level**						
Junior Secondary	0.000	Reference	Reference	0.000	Reference	Reference
Senior Secondary	0.122	0.026	0.014–0.229	0.053	0.382	-0.066- 0.172
**Location**						
Rural	0.000	Reference	Reference	0.000	Reference	Reference
Urban	-0.128	0.013	-0.228- -0.027	-0.141	0.006	-0.240–0.041
**Work for pay**						
No	0.000	Reference	Reference	0.000	Reference	Reference
Yes	-0.007	0.907	-0.137–0.121	-0.079	0.217	-0.206- 0.046
**Live with parent**						
No	0.000	Reference	Reference	0.000	Reference	Reference
Yes	0.015	0.883	-0.192–0.223	0.065	0.504	0.126–0.257

Table 4 shows predictors of adolescents’ perceived value towards SRH services. Being in the intervention group increases adolescents’ perceived value for SRH services by 20% (β = 0.197, CI: 0.141–0.253) as compared to the non-intervention group. On the average, participants in senior secondary school had about 9% higher perceived value scores toward sexual and reproductive health services compared to the participants in junior secondary school (β = 0.089, CI: 0.019–0.160). Being aware of available SRH services increases adolescents’ perceived value for SRH services by 19% (β = 0.192, CI: 0.425–0.721). On the average, participants who worked for pay had an 8% lower perceived value score toward SRH services compared to the perceived score among participants who do not work for pay (β= -0.079, CI: -0.156 - -0.002).

**Table 4:** Linear regression of socio-demographic correlates of adolescent’s perceived value toward sexual and reproductive health services

Variable	Univariable regression			Multivariable regression		
	Crude Coefficientβ	P-value	Confidence interval	Adjusted Coefficient (Adjusted β)	P-value	Confidence interval
**Study group**						
Non-intervention group	0.000	Reference	Reference	0.000	Reference	Reference
Intervention group	0.204	< 0.0001	0.148–0.260	0.197	< 0.0001	0.141–0.253
**Gender**						
Male	0.000	Reference	Reference	0.000	Reference	Reference
Female	-0.019	0.547	− 0.082–0.043	-0.034	0.265	-0.096- 0.026
**Age**	0.038	< 0.0001	0.020–0.057	0.016	0.141	-0.005- 0.039
**Educational Level**						
Junior Secondary	0.000	Reference	Reference	0.000	Reference	Reference
Senior Secondary	0.131	< 0.0001	0.070–0.191	0.089	0.013	0.019–0.160
**Location**						
Rural	0.000	Reference	Reference	0.000	Reference	Reference
Urban	-0.035	0.241	-0.094- 0.023	-0.051	0.086	-0.109-0 0.007
**Work for pay**						
No	0.000	Reference	Reference	0.000	Reference	Reference
Yes	-0.055	0.159	-0.133–0.021	-0.079	0.044	-0.156- -0.002
**Live with parent**						
No	0.000	Reference	Reference	0.000	Reference	Reference
Yes	-0.019	0.764	-0.144- 0.106	0.023	0.703	-0.095- 0.141
**Aware of available SRH services**	0.189		0.173–0.205	0.192	< 0.0001	0.425–0.721

Table 5 shows the predictor of societal support for adolescents’ utilisation of SRH service. On the average, participants who worked for pay had about 10% higher parental/community support score for SRH service utilization as compared to parental/community support score among participants who do not work for pay (β = 0.095, CI: 0.003–0.185).

**Table 5:** Linear regression analysis of socio-demographic correlates of parental/community support to adolescents’ utilisation of SRH services

Variable	Univariable regression			Multivariable regression		
	Crude Coefficientβ	P-value	Confidence interval	Adjusted Coefficient (Adjusted β)	P-value	Confidence interval
**Study group**						
Non-intervention group	0.000	Reference	Reference	0.000	Reference	Reference
Intervention group	0.006	0.863	-0.060–0.071	-0.007	0.819	-0.074- 0.058
**Gender**						
Male	0.000	Reference	Reference	0.000	Reference	Reference
Female	-0.016	0.656	-0.087- 0.054	0.010	0.790	-0.063- 0.084
**Age**	0.021	0.044	0.001–0.042	0.010	0.442	-0.015- 0.036
**Educational Level**						
Junior Secondary	0.000	Reference	Reference	0.000	Reference	Reference
Senior Secondary	0.071	0.040	0.003 - 0.139	0.053	0.203	-0.029- 0.136
**Location**						
Rural	0.000	Reference	Reference	0.000	Reference	Reference
Urban	-0.005	0.865	-0.071- 0.060	0.014	0.694	-0.056- 0.084
**Work for pay**						
No	0.000	Reference	Reference	0.000	Reference	Reference
Yes	0.083	0.053	-0.001- 0.167	0.095	0.042	0.003–0.185
**Live with parent**						
No	0.000	Reference	Reference	0.000	Reference	Reference
Yes	-0.054	0.448	-0.194- 0.086	-0.048	0.500	-0.191- 0.093

## Discussion

The study compared the experiences of adolescents that had received targeted adolescent SRH interventions and those that did not and evaluated the determinants of awareness, perceived value, and societal support for SRH service utilisation among secondary school adolescents in Ebonyi state, Nigeria. The study findings revealed that a low proportion of the adolescents were aware of available SRH services at health facilities and communities. However, more adolescents in the intervention than in the non-intervention group reported community support and perceived that adolescents need and should be provided with SRH services. As awareness of SRH services can enhance enforcement of the child rights act regarding the right to health and well-being, [22] intensifying awareness of the SRH programme is worthwhile to promote and sustain the value and support for its utilisation.

Only about half of the adolescents were aware of the types of SRH services provided at the health facilities/communities and what days and times health services are provided in the intervention group, and less than a fifth of the non-intervention groups. This finding corroborates a study on access and utilisation of reproductive health services conducted in Kaduna, Nigeria, [27] and Tanzania, [28] where poor awareness was reported. An earlier survey reported that awareness of sources and available SRH services remains low in African countries, with fewer than half of adolescents knowing where to obtain SRH services. [15] Contrary to our findings, a study of awareness of SRH services in Ghana [29] and among female secondary school students in Ethiopia [30] indicated that about seven out of ten students were aware. The higher awareness in the later study may be connected to female study subjects. Females are more prone to more sexual risk because of their anatomy and hence, may be more interested in sexual and reproductive health issues. [29]

Although the awareness of SRH services was generally poor, a significantly higher proportion of adolescents that had received SRH intervention were aware compared to those that had not. Adolescents in the intervention group were 38% more aware of SRH services compared to the non-intervention group. This finding is comparable to a school-based intervention study which reported higher awareness among students in the intervention group than in the control group. [31] The higher awareness among the intervention group may be connected to the multi-faceted intervention. The school-based activities and adolescent involvement in the design and implementation of the intervention, including peer education, may have contributed to better exposure to SRH information and increased awareness. Youth participation in the design and development of programmes and policies led to better sexual and reproductive health outcomes. [32] This finding highlights the importance of intensifying SRH programmes in all schools.

Awareness of SRH services was also found to be associated with age and location of residence. For every year increase in age, there was a 4% increase in awareness of available SRH services This corroborates a study finding on SRH among in-school adolescents, which indicated that age was significantly associated with awareness. [33] Furthermore, living in an urban area decreases awareness of available SRH services by 14% compared to living in rural areas. However, an earlier study among secondary school students in Ethiopia reported that place of residence has no association with awareness. [30] It is, therefore, crucial to remember that adolescents are a diverse group with varying needs while selecting intervention implementation approaches. Implementation strategies should be tailored not only to the context but according to the specific needs of different subgroups of adolescents.

Adolescents value sexual and reproductive health services. About eight out of ten adolescents in both the intervention and non-intervention groups perceived that adolescents need SRH services and that reproductive health services should be provided. This is not surprising as the study participants are in-school adolescents who have the opportunity of being exposed to the sexual and reproductive health curriculum and to appreciate the importance of SRH. A significantly higher proportion of adolescents in the intervention group than the non-intervention group perceived SRH as valuable, consistent with findings of an earlier study. [34] As programmes that address user and service-provision issues have been known to be successful and valued, [35] the high value for SRH services noted in this study may be linked to interventions that applied a similar approach. The intervention for the study combined different strategies that addressed user issues by including adolescents in the planning and implementation, service provision from teachers and peer educators through training and collaborative learning, and demand generation and buy-in from the community. Our finding also compares with a recent review which found that programs that trained personnel was effective in promoting SRH services among adolescents. [36]

Furthermore, the study found that an increase in educational levels increases the value of SRH services, consistent with a study on secondary school students in Ethiopia. [20] This is because adolescents at higher educational levels are more likely to engage in discussion with teachers and parents about SRH issues compared to those at the lower level and so more aware, [20] hence, corroborating our finding. Ideally, SRH information should be provided to all adolescents and designed to meet the needs of different adolescent subgroups across varying contexts. This includes increasing access to SRH-friendly services that respect adolescents’ rights to health services and privacy which is crucial in ensuring that adolescents seek and receive these services.

This study showed that more than two third of the adolescents in both the intervention and non-intervention groups reported having the support of their parents/guardians and adults in the community to utilise SRH services. Contrary to our findings, less than a third of young people in a study conducted in Ethiopia perceived that their parents do not positively respond to their SRH needs. [20] The positive disposition of parents/guardians and community members to adolescents’ utilisation of SRH services may be attributed to the implemented interventions and well as, the COVID-19 pandemic. This is because, in addition to the interventions implemented in schools and communities, sensitisation on health issues, including adolescent SRH needs and rights, was prioritised during the pandemic with emphasis on community buy-in and participation, which may have influenced their attitude.

A significantly higher proportion of adolescents in the intervention group than in the non-intervention group experienced parental and community support to utilise SRH services. Our finding corroborates a recent review which found that programmes that trained healthcare providers to respond appropriately to adolescents’ needs achieved community acceptance and demand generation effective in promoting access and uptake of SRH services among adolescents. [36]

The main limitation of this study is its focus on adolescents aged 13–19 years, leaving out younger adolescents. Thus, the study cannot be generalised to all secondary school adolescents. There is a need to focus on the specific needs of younger adolescents aged 10–12, for which sex education is a high priority. This is because, while this age group is less likely to be sexually active and need sexual and reproductive health services than older adolescents, those needing services are especially likely to be underserved by providers and neglected. It is impossible to guarantee that adolescents provide honest answers to questions, especially with sensitive issues such as sexual and reproductive health. Thus, the data may be subject to social-desirability bias, where respondents may have felt obliged to report positively on the SRH issues. However, the research assistants were well-trained to disassociate themselves from the implementation team and probe for all positive and negative opinions. There could be an intraclass/intraschool correlation among the participant, which may have limited the findings. Multi-level regression modelling is recommended in future studies.

## Conclusions

Adolescents have a high value for SRH services and parental/community support for SRH service utilisation which is higher among the intervention group and influenced by social and demographic factors. Stakeholders should intensify awareness, build capacity within existing systems to address adolescent challenges and institutionalise comprehensive sex education in schools and communities to target various categories of adolescents and maximise reach.

## Data Availability

The dataset generated and/or analysed during this study is available and can be obtained from the corresponding author upon request, as well as any other material needed.

## List of abbreviations

AFHS: Adolescent Friendly Health Services
ASRH: Adolescent Sexual and Reproductive Health
LGA: Local Government Area
JSS: Junior Secondary School
SRH: Sexual and Reproductive Health
WHO: World Health Organization
SS: Senior Secondary School
